# Psychiatric hospitalization among offenders: a retrospective study in the acute psychiatric ward in Monza, Italy

**DOI:** 10.1192/j.eurpsy.2023.925

**Published:** 2023-07-19

**Authors:** M. Provenzi, L. M. Affaticati, G. Carrara, C. L. Di Forti, D. Viganò, M. Clerici

**Affiliations:** 1 Psychiatric Department, ASST Monza, Italy; 2 Department of Medicine and Surgery, University of Milano Bicocca, Monza, Italy

## Abstract

**Introduction:**

The closure of forensic psychiatric hospitals and the conversion to a residential model of care based on secure residential units in the community (REMS) has made Italy the first and only country in the world to have followed the principles of the deinstitutionalization movement. Following the reform, several management issues have emerged, such as the creation of long waiting lists for admission to REMS. Improper hospitalization in Acute Psychiatric Units (SPDC) has often been used to address this issue. In addition, the handover of inmates’ care to Mental Health Departments (DSMD’s) has posed further challenges. To date, the field has received little attention from international literature.

**Objectives:**

Description and analysis of a sample of offender inpatients hospitalized in an acute psychiatric unit.

**Methods:**

We conducted a retrospective study including male offenders admitted to the SPDC of San Gerardo Hospital (ASST Monza), between January 2007 and September 2022. Data analysis was performed by using SPSS.

**Results:**

120 male offenders were included for a total of 204 admissions. 98 offenders (81.7%) were hospitalized once. We observed an absolute (N=1; N=30) and percentage (0.2%;12%) increase in the number of hospitalized offenders per year during the time period under study. Jail was the main provenance in the sample (46.6%), followed by residential care facilities (27%) and the psychiatric observation unit (ROP) of Monza’s jail (10.8%). The two most prevalent diagnoses were personality disorders (37.5%) and psychosis (39.2%). In addition, 66 subjects (55%) had a history of substance abuse. The average duration of hospitalization was 19.45 days; it increased to 77 days for inpatients waiting to be transferred to REMS. Hetero-aggressive behavior as the reason for admission was associated with longer hospitalization (p=0.031), while attempted suicide correlated to shorter hospital stay (p=0.032). Out of the 55 offenders who attempted suicide, 41 came from jail (74.5%). Finally, longer hospitalizations were associated with an increased number of adverse events (p=0.001).

**Conclusions:**

Psychiatric hospitalizations of offenders have increased over the last years. This population tends to require longer hospital stays (regional average of SPDC hospitalization in Lombardy: 14 days), which are even lengthier for inpatients destined to REMS. Longer hospitalizations exert a large burden on DSMD’s and impact the general health of patients, exposing them to a higher risk of adverse events. Further studies are needed to confirm our findings and to develop better strategies for the management and care of offender patients.

**Disclosure of Interest:**

None Declared

